# Development and Application of an UHPLC-MS/MS Method for Comparative Pharmacokinetic Study of Eight Major Bioactive Components from Yin Chen Hao Tang in Normal and Acute Liver Injured Rats

**DOI:** 10.1155/2018/3239785

**Published:** 2018-11-01

**Authors:** Yun Wang, Xinrui Xing, Yan Cao, Liang Zhao, Sen Sun, Yang Chen, Yifeng Chai, Si Chen, Zhenyu Zhu

**Affiliations:** ^1^Hebei Institute for Drug Control, No.16 Fuqiang Street, Shijiazhuang 050011, China; ^2^School of Pharmacy, Second Military Medical University, No. 325 Guohe Road, Shanghai 200433, China; ^3^Department of Pharmacy, Eastern Hepatobiliary Surgery Hospital, Shanghai 200438, China; ^4^Postdoctoral Research Workstation, 210th Hospital of the Chinese People's Liberation Army, Dalian 116021, China

## Abstract

Yin Chen Hao Tang (YCHT) is one of the most famous hepatoprotective herbal formulas in China, but its pharmacokinetic investigation in model rats has been rarely conducted. In this study, the hepatic injury model was caused by intraperitoneal injections of carbon tetrachloride (CCl_4_), and YCHT was orally administered to the model and normal rats. An ultrahigh performance liquid chromatography–tandem mass spectrometry (UHPLC–MS/MS) method was established to analyze the plasma pharmacokinetics of eight major bioactive ingredients from YCHT in both the normal and liver injured rats. The calibration curves presented good linearity (r > 0.9981) in the concentration range. The relative standard deviation (RSD%) of inter- and intraday precision was within 9.55%, and the accuracy (RE%) ranged from -10.72% to 2.46%. The extraction recovery, matrix effect, and stability were demonstrated to be within acceptable ranges. The lower limit of detection (LLOD) and lower limit of quantitation (LLOQ) were around 0.1 ng/mL and 0.5 ng/mL, respectively, which were much lower than those in other related researches. Results reveal that there are significant differences in the pharmacokinetics of scoparone, geniposide, rhein, aloe-emodin, physcion, and chrysophanol in hepatic injured rats as compared to those in control except for scopoletin and emodin. Our experimental results provide a meaningful reference for the clinical dosage of YCHT in treating liver disorders, and the improvement of LLOD and LLOQ can also broaden the range of our method's application, which is very suitable for quantitating these eight compounds with low levels.

## 1. Introduction

Yin Chen Hao Tang (YCHT) is one of the most famous herbal formulas recorded in Shanghanlun of Zhongjing Zhang in China. It contains three component herbs: Artemisia capillaris Thunb (Yinchen), Gardenia jasminoides Ellis (Zhizi), and Rheum Palmatum L (Dahuang). YCHT has long been applied in clinical practice in treating liver diseases, such as jaundice and hepatic fibrosis [1–3]. The main constituents of YCHT are coumarins from Yinchen, iridoid glycosides from Zhizi, and anthraquinones from Dahuang (supplementary Figure S1) [2]. And previous study demonstrated that the coumarin components from Yinchen, such as scoparone and scopoletin, were the ingredients which conveyed the hepatoprotective and anti-inflammatory effect [4, 5]. In addition, geniposide, rhein, aloe-emodin, chrysophanol, emodin, and physcion, the major components in Zhizi and Dahuang, are also active ingredients against liver injury [6, 7]. These main active ingredients have been identified in the rats' serum after oral administration of YCHT [8, 9].

To date, several publications are available on the pharmacokinetic properties of the above 1-3 active components in YCHT in healthy rats [2, 9, 23–25]. However, researchers have presented that chemicals' pharmacokinetic profile may be influenced by liver disease, such as liver fibrosis [26, 27] and nonalcoholic fatty liver disease [28]. Studies also have illustrated that liver disease will affect the pharmacokinetics of the drug in human [29]. As YCHT has long been applied clinically for treating liver diseases, it would be more meaningful to conduct pharmacokinetic research of YCHT in model organisms. A recent pharmacokinetic study demonstrated the differential absorption and distribution process of scoparone in alpha-naphthylisothiocyanate induced experimental hepatic injured rats as compared to in normal rats [30]. However, only one compound in YCHT was studied.

In order to comprehensively evaluate and compare the pharmacokinetic profile of YCHT in normal and liver injured rats, we aim to develop a mass spectrometry based method for rapid quantitation of eight major bioactive ingredients of YCHT in rat plasma after oral administration of YCHT. Until now some analytical methods have been proposed for quantitating 1-3 components in YCHT in vivo, including high-performance liquid chromatography (HPLC) with UV detection [2, 23–25, 31, 32], ultra-performance liquid chromatography (UPLC) with UV detection[30], and ultra-performance liquid chromatography-electrospray ionization/quadruple-time-of-flight mass spectrometry (UPLC-ESI/Q-TOF–MS/MS)[8, 9, 33]. However, above methods showed the shortage of long analysis time and/or low sensitivity and thus are not appropriate for determining eight active ingredients of YCHT in plasma. LC–MS/MS in dynamic multiple reaction monitoring (DMRM) mode, with impressive sensitivity and inherent selectivity, has acted as a gold standard for accurate multianalyte quantitation of large samples to supply high-quality data from complex systems [34]. And our previous studies also proved its advantages [35, 36].

In this study, a fully validated UHPLC–MS/MS method was established and successfully used to the measurement and pharmacokinetic investigation of eight representative active components in YCHT in both normal and live injured rats. This study will provide a meaningful basis for investigating a clinical dosage regimen of YCHT in treating liver-related diseases.

## 2. Experimental

### 2.1. Materials and Reagents

Scoparone, scopoletin, geniposide, rhein, aloe-emodin, emodin, chrysophanol, and physcion were purchased from Shanghai Standard Biotech Co., Ltd. (Shanghai, China). Glibenclamide and wogonin, the internal standards (IS) in positive and negative mode, were obtained from China's National Institute for the Control of Pharmaceutical and Biological Products (Beijing, China). The compounds' purities were above 98% determined by HPLC/UV.

*Artemisia capillaris* Thunb. (collection in Anhui, China),* Gardenia jasminoides* Ellis (collection in Hubei, China), and* Rheum Palmatum* L. (collection in Gansu, China) were purchased from Shanghai Dekang Medicine Corp. (Shanghai, China). And these herbs were authenticated by Lianna Sun (Department of Pharmacognosy, School of Pharmacy, Second Military Medical University, Shanghai, China).

LC/MS-grade methanol was obtained from Sigma-Aldrich (St. Louis, MO, USA). The ultrapure water was prepared with a Milli-Q water purification system (Millipore, Bedford, MA, USA) throughout the study. HPLC-grade acetonitrile was obtained from Merck (Darmstadt, Germany) and formic acid was purchased from Fluka (Buchs, Switzerland). Carbon tetrachloride (CCl_4_) was supplied by Jiangsu Qiangsheng Chemical Co., Ltd. (Jiangsu, China). Olive oil (Oliver grade) was purchased from Branch office of Shanghai of Olis Olive Oil Bloc (Catalonia, Spain). All other reagents are analytical level or better.

### 2.2. Instrumentation and UHPLC–MS/MS Analytical Conditions

The quality control (QC) and plasma samples were analyzed by an Agilent 6470 triple quadrupole LC–MS system (Agilent Technologies, Inc., Santa Clara, CA, USA), which consists of an Agilent 1290 Infinity II system connected to a triple quadrupole MS analyzer with an Agilent Jet Stream technologies electrospray ionization (ESI) interface. The Agilent 1290 LC system is equipped with degasser (G7116B), high speed pump (G7120A) and multisampler (G7167B). The samples (2 *μ*L) were injected into the LC system. A MassHunter workstation was applied for LC–MS control and data acquisition.

Chromatographic separation was conducted with a CAPCELL PAK ADME (2.1 mm × 100 mm, 3 *μ*m; Shiseido Co., Ltd., Tokyo, Japan). The mobile phase included 0.1% formic acid (A) and methanol (B), and the flow rate was 0.3 mL/min. Iridoid glycosides and anthraquinones were detected in the isocratic elution, which was kept 80% B for 5.5 min in the negative mode. The others were separated in the positive mode with another gradient program, which was maintained at 45% B for 0.5 min, then increased to 95% B in 2 min, and maintained at 95% B until 4.5 min, and the post time is 2 min.

The optimal parameters for the ESI interface were as follows: nebulizer, 45 psi; dry gas, 10 L/min; dry temp, 350°C; sheath gas, 11 L/min; sheath gas temp, 350°C; capillary voltage, 4000 V (in the positive mode) and 3500 V (in the negative mode); and delta EMV, 200 V. The MS parameters for iridoid glycosides and anthraquinones were improved in the negative mode, and the MS parameters for the other analytes were improved in positive mode. The precursor-to-product ion transitions subjected to DMRM were listed in Table 1.

### 2.3. Preparation of YCHT Extract

The preparation method of YCHT was from the original description in “Shanghan Lun” and had been applied in many researches [8, 37, 38]. The detailed procedures were as follows. First, Yinchen 720 g was decocted with 14.4 L deionized water, after the solution volume was reduced to half, and the mixed powdered samples of Zhizi 360 g and Dahuang 240 g was added. The solution was maintained boiling for 10 min and filtered through six-layer bandage. Next, 7.2 L deionized water was put in the residues and refluxed for additional 30 min and then filtered with six-layer bandage. The two batches of filtrates were mixed and condensed under vacuum into 440 mL (equal to 3 g herb mL^−1^) and then stored in 4°C in refrigerator before intragastric administration to rats. The quantitative analysis of the main bioactive components in YCHT extract was determined by HPLC-DAD before the pharmacokinetic experiment. The contents of scoparone, scopoletin, geniposide, rhein, aloe-emodin, chrysophanol, physcion, and emodin were 0.5047, 0.0243, 0.8582, 0.4931, 0.1328, 0.1122, 0.0290, and 0.0416 mg/mL, respectively.

### 2.4. Preparation of Standard Solutions and QC Samples

Each standard's stock solutions was generated by dissolving substances in dimethyl sulfoxide and stored at −20°C in the dark. And working standard solutions with mixed standards were generated by diluting a stock-standard mixture with acetonitrile. The concentrations of scoparone, scopoletin, geniposide, aloe-emodin, rhein, chrysophanol, emodin, and physcion in the working standard solutions were in the range of 0.510–2040.0, 0.502–50.2, 0.516-2064.0, 0.532-106.4, 0.540-2160.0, 0.500-100.0, 0.525-52.5, and 0.512–51.2 ng/mL, respectively. The stock solutions of glibenclamide and wogonin were mixed and diluted with acetonitrile to generate a ISs working solution with 100.1 ng/mL glibenclamide and 100.3 ng/mL wogonin. All standard working solutions were stored at 4°C.

One hundred *μ*L mixture working solutions in the centrifuge tube was evaporated to dryness, and the plasma calibration standards were prepared by adding 100 *μ*L blank rat plasma into above tube. QC samples, with LLOQ, low, middle, and high plasma concentrations, were prepared like the calibration standards.

### 2.5. Animal Grouping and Model Preparation

All animal experiments were conducted at the Animals Laboratory Centre of the Second Military Medical University (Shanghai, China) based on the Committee's guidelines on the Care and Use of Laboratory Animals in China. Twelve male 6 weeks' Sprague-Dawley rats (180 ± 20 g) were purchased from the Shanghai Laboratory Animal Co. (SLAC, Shanghai, China) and were acclimatized for at least 7 days before the experiments. Every four rats were housed in a standard cage with 12 h light/dark cycle (lights on at 7:00 am). Purified water and commercial diet were supplied freely. The temperature of the animal rooms was set to 20-25°C and the relative humidity was 50-70%.

The animals were randomly divided into the control group (n = 6) and the model group (n = 6): the model rats, i.e., acute liver injury rats, were induced by intraperitoneal injections of CCl_4_ diluted with olive oil (1:1, v/v) at a dose of 1.5 mL/kg.bw every day for three days, and the rats in the control group were administered with olive oil.

After finishing the administration of CCl_4_, serum samples were collected and subjected to biochemical analysis. Serum concentrations of aspartate aminotransferase (AST) and alanine aminotransferase (ALT) were determined by routine methods using the Hitachi 7600 automatic analyzer (Hitachi High-Technologies Corporation, Tokyo, Japan).

The rats' livers were cut off after pharmacokinetic experiment and fixed in 10% formaldehyde solution. Liver tissues were then embedded in paraffin wax, cut and stained with hematoxylin-eosin (HE). And the histopathological changes were examined under the microscope (Olympus DX45, Japan). Olympus DP72 was used to take the images at original magnification of 100X.

### 2.6. Plasma Sample Preparation

After being thawed at room temperature, 100 *μ*L plasma was put in a 1.5 mL centrifuge tube, and then 100 *μ*L IS solution and 300 *μ*L acetonitrile was added. After the mixture was vortexed for 1 min, it was centrifuged at 12000 rpm for 10 min at 4°C to precipitate the protein. Then the supernatant was transferred to another clean centrifuge tube, and evaporated to dryness under nitrogen stream, the residue was reconstituted in 50 *μ*L methanol-water (1:1, v/v). After centrifuging at 12000 rpm for 10 min at 4°C again, a 2 *μ*L aliquot of supernatant was injected for LC–MS/MS analysis.

### 2.7. Method Validation

Following the FDA guidance for industry [39], the method validation process was carried out by evaluating selectivity, matrix effect, linearity, extraction recovery, accuracy and precision, and stability parameters.

The method's selectivity was evaluated by comparing the chromatograms of six blank plasma samples from six different rats with the standard plasma samples spiked with mixed-compound solution and ISs to check for any potential interfering peaks of the analytes and ISs.

The matrix effect was investigated with different blank matrices from different rats. Matrix factor (MF) was obtained as the ratio of the peak responses of analyte and IS spiked matrices to a pure standard solution at equivalent concentration (low, middle, and high QC levels). The IS-normalized MF was obtained by the following formula: IS-normalized MF (%) = MF_analyte_/MF_IS_ × 100%. The relative standard deviation (RSD) of the IS-normalized MF from six individual matrices should be less than 15%. The experiments were conducted in six different batches at three QC levels.

The method's linearity was assessed by six-point calibration curves. The calibration curves were formed by weighed (1/*x*) least-squares linear regression model of the peak-area ratios of each analyte to IS (*y*) versus the nominal analyte concentrations (*x*).

The inter-day and intra-day accuracy and precision were assessed by analyzing QCs at three concentration levels (low, middle, and high) in six replicates on three consecutive days. The accuracy was obtained by calculating the ratio of the measured concentration to its nominal level. The RSD and the relative error (RE) of each QC concentration reflected the method's precision and accuracy. For intra-day precision, the measurements were performed six replicates one day. Inter-day precision can be gotten from different sample batches on three consecutive days.

The extraction recovery was calculated using the ratio of the analytes' peak area in extracted samples with the analytes' average peak area in samples where the analyte was spiked after extraction. The analytes from plasma at low, middle and high three QC levels were detected in six replicates.

The test analytes' stability was assessed in rat plasma by evaluating quintuplicates of QCs at low, middle and high levels. The samples' stability was analyzed under the following conditions: (1) kept at room temperature for 2 h; (2) kept at -80°C for 15 days; (3) after three freeze-thaw cycles at -80°C; (4) postpretreated plasma sample in an autosampler (4°C) for 24 h. The responses were detected in triplicate.

The LLOD is the amount of drug in plasma after sample clean up corresponding to 3 times the baseline noise ratio (S/N > 3). And the LLOQ is the analyte concentration that can be quantified with ± 20 % accuracy and precision. Its signal should be at least 10 times as compared to blank (S/N > 10).

### 2.8. Pharmacokinetic Studies

The validated method was used for the pharmacokinetic study. Standard diet was withheld over night before oral administration of YCHT, but with free access to water. And YCHT (60 g/kg) was administered to the rats by gastric perfusion, equivalent to 10.09 mg/kg of scoparone, 0.49 mg/kg scopoletin, 17.16 mg/kg of geniposide, 9.86 mg/kg of rhein, 2.66 mg/kg of aloe-emodin, 2.24 mg/kg of chrysophanol, 0.83 mg/kg of emodin, and 0.58 mg/kg of physcion.

A series of blood samples (0.3 mL) were collected from the rats' postorbital venous plexuses at 0 (pre-dose), 5, 10, 15, 30, 45, 60, 90, 120, 240, 480, 600, 720 and 1440 min, and put into heparinized tubes. The heparinized plasma was immediately centrifuged to separate at 3000* g* for 10 min and stored at −80°C before experiments. The analytes' concentrations in plasma were determined by LC–MS/MS methods described above.

### 2.9. Statistical Analysis

The plasma concentrations were expressed as arithmetic mean ± standard deviation (SD). And the mean concentration–time curves were calculated and plotted by DAS (version 3.0, Bio Guider Co., Shanghai, China), and the PK parameters consist of elimination half-life (t_1/2_), area under the plasma concentration-time curve (AUC), and mean residence time (MRT). The maximum plasma concentrations (C_max_) and corresponding peak time (T_max_) were obtained from the observed data directly. The relative bioavailabilities (Fr) in this study were all compared with control group, which was calculated as: Fr = (AUC_model_/AUC_control_) *∗*100%. Student's t-test in SPSS V22.0 (SPSS, Chicago, USA) was used to compare the difference between two groups at the same phase.* P*<0.05 represented statistical significance.

## 3. Results and Discussion

### 3.1. UHPLC–MS/MS Analytical Method Validation

Different compositions of mobile phase were investigated to obtain optimal response, good peak shape and suitable retention time. Each paired combination of acetonitrile, methanol, and 0.1% formic acid in water was tested. As a result, 0.1% formic acid in water and methanol was used as the best solvent system. In addition, both positive and negative modes were tested in the MS condition. And due to the nature of each compound, the analysis was finished in two modes with two methods, respectively.

### 3.2. Validation Procedures

#### 3.2.1. Specificity and Selectivity

**Figure 1:**
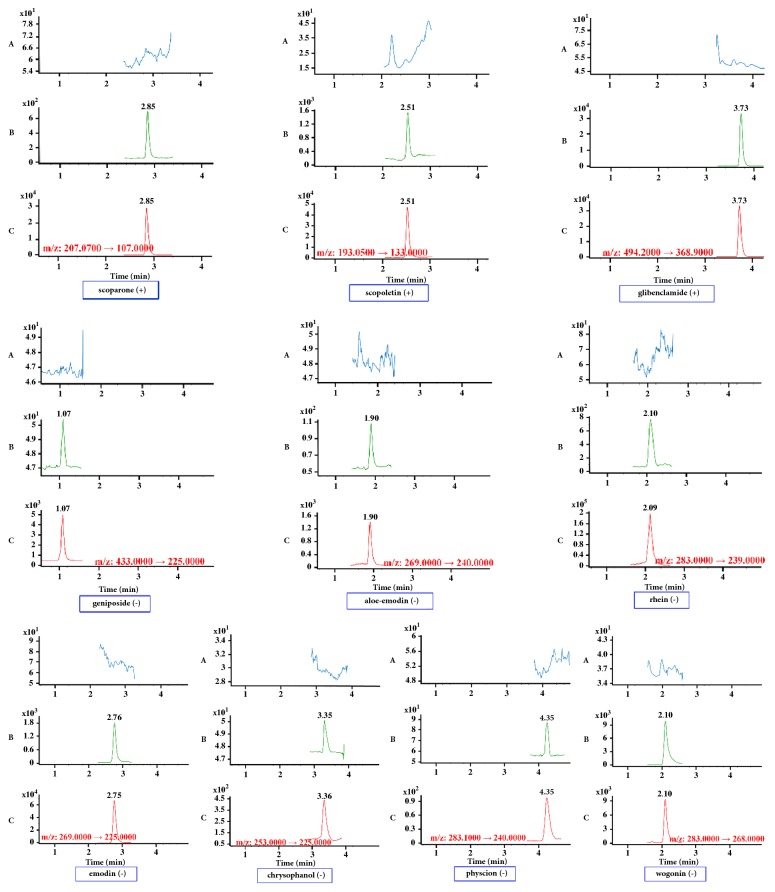
*Representative DMRM chromatograms for scoparone, scopoletin, geniposide, aloe-emodin, rhein, emodin, chrysophanol, physcion, and their internal standards glibenclamide and wogonin in rat plasma samples*. Panel A: a blank plasma sample; panel B: a blank plasma sample spiked with bioactive compounds and ISs at the LLOQ; panel C: a rat plasma sample collected at 30 min after drug administration.

Iridoid glycosides and anthraquinones exhibited favorable sensitivity in negative ion mode, while other analytes were more sensitive in positive ion mode. Thus two sensitive ISs were chosen in positive and negative modes, respectively. Glibenclamide and wogonin, the ISs selected in present experiment, could meet the requirement. As is shown in Figure 1, no interfering endogenous substance was observed at the retention times of all ingredients and ISs.

#### 3.2.2. Linearity and LLOQ

Calibration curves, correlation coefficients, linear ranges, LLOD, and LLOQ of the 8 constituents were showed in Table 2. A 1/*x*-weighting scheme was applied for validation and analysis in plasma. All 8 constituents showed good linearity within selected concentration ranges. Their linear correlation coefficients (r) were all better than 0.9981.

#### 3.2.3. Precision and Accuracy

Accuracy and precision of our method were validated by assaying the QC samples in plasma at three concentration levels. The intra- and inter-day accuracy and precision for all test compounds are shown in supplementary Table S1. In this assay, the RSDs for intra-day precision were 1.51-9.55% while inter-day precisions were 1.57-8.88%. The accuracy ranged from -10.72% to 2.46%. Thus our method was accurate and precise.

#### 3.2.4. Recovery and Matrix Effect

The results of matrix effect and extraction recovery are summarized in supplementary Table S2. It indicated that the signal response of each component and ISs in different sources of blood samples (LQC, MQC, and HQC) is stable, which can meet the biological sample quantitative requirements. According to the bioanalytical method validation guideline, the average recoveries of 8 analytes are acceptable.

#### 3.2.5. Stability

The summary of stability studies is presented in supplementary Table S3. The data indicated that the RSD values of long-term stability, short-term stability, freeze–thaw stability, and postpretreated stability of analytes for 24 h for LQC, MQC, and HQC (n = 5) were within 11.00%. All 8 analytes were stable in conditions likely to encounter, such as sample preparation, sample detection, and storage.

### 3.3. Biochemical and Physiological Parameters in the Experimental Rats

**Figure 2:**
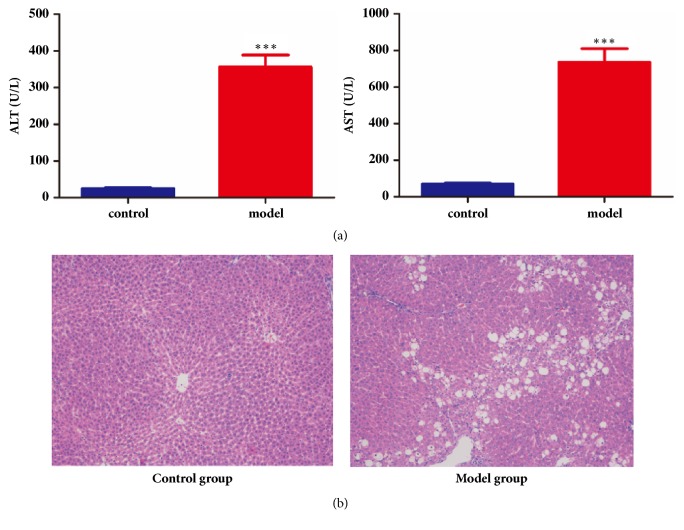
*Biochemical and physiological parameters in the experimental rats*. (a) Biochemical parameters of normal and model rats. (b) Representative photomicrographs of histopathological studies of livers stained with hematoxylin and eosin (100 ×).

ALT, AST, and pathological section of model and control rats were determined. As shown in Figure 2(a), the CCl_4_ intoxication caused a severe hepatic injury by tremendous enhancement of ALT and AST levels (p ≤ 0.01).

HE staining in Figure 2(b) also demonstrated that three days' CCl_4_ administration caused hepatic injury in rat. There are large areas of inflammatory infiltration in model rats. And while compared to the normal rats, the scattered white areas observed on the livers of CCl_4_ intoxication rats indicated necrotic changes and fatty depositions. With the combination of above two standards, a conclusion can be made that the rats in the model group were liver-injured.

### 3.4. The Pharmacokinetic Characteristics of the Active Components

**Figure 3:**
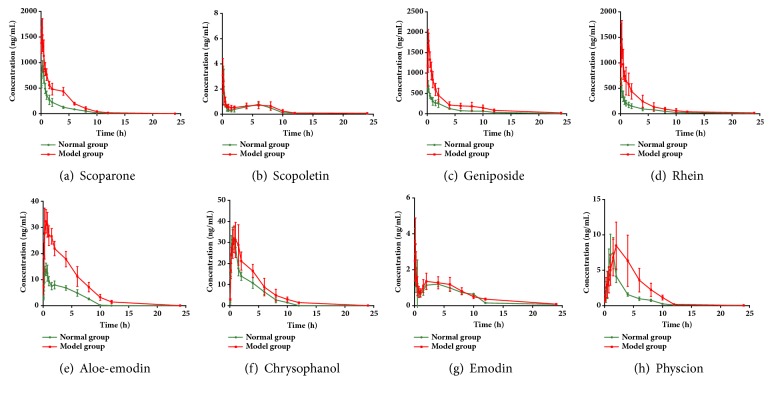
Mean concentration-time profiles of (a) scoparone, (b) scopoletin, (c) geniposide, (d) aloe-emodin, (e) rhein, (f) emodin, (g) chrysophanol, and (h) physcion based integrated concentration in control and model rat plasma after oral administration of YCHT (n = 6).

The validated analytical method was successfully applied to investigate the pharmacokinetic profiles of 8 major bioactive constituents in the normal and liver injured rats' plasma after a single oral administration of YCHT. Their distribution and elimination processes are fitted into the noncompartment model. And the concentration versus time curves for all the compounds in normal and the hepatic injured rats are shown in Figure 3 and the pharmacokinetic data is shown in Table 3.

After YCHT was orally administered, two coumarins were detected in the normal and liver injured rats' plasma. The C_max_ of scoparone in the model rats was statistically higher than that in normal rats. In addition, the AUC value of scoparone in the model rats was also approximately 2-fold higher than that in normal rats. It indicated that scoparone exhibited different pharmacokinetic characteristics under liver injury state compared to normal state. Unlike scoparone, scopoletin's pharmacokinetic characteristics indicate no significant differences in hepatic injured rats compared to in normal rats (Figure 3(b)). And its C_max_, t_1/2_, etc. are quite consistent under physiological and pathological status (Table 3).

Geniposide's pharmacokinetic characteristics are quite similar to that of scoparone in both normal and model rats (Figure 3(c)). Therefore, the plasma concentrations of geniposide are detectable at all sampling time in both groups. T_max_ of geniposide in the normal and model rats are 0.33±0.13 h and 0.58±0.20 h, respectively (Table 3), which indicated a statistically delay in disease state. Moreover, in the model rats, the C_max_ value (1863.84±108.53 ng/mL) of geniposide is significantly higher in comparison with the normal group (751.37±47.72 ng/mL, P < 0.01). The AUC_0–*∞*_ value of geniposide in the model rats is 5000.36±755.13 *μ*g /L*∗*h, almost two times higher than that in the normal group (1940.63±210.72 *μ*g /L*∗*h). And this may lead to a significant accumulation of geniposide* in vivo*.

The mean blood concentration-time profiles of these five anthraquinones in Dahuang are illustrated in Figure 3. As shown in Table 3, the AUC_0–*∞*_ of the liver injured rats were higher than that of normal rats. And the obvious lower CLz/F compared with normal rat groups were observed, which means the metabolic rate of these compound slows down under pathological conditions. There is a second peak of emodin. These bimodal patterns may be related to its specific metabolism and enterohepatic circulation. However, emodin's pharmacokinetic characteristics present no significant differences in liver injured rats as compared to in normal rats (Figure 3(g)). And its pharmacokinetic parameters, such as C_max_, T_max_, and t_1/2_, CLz/F, are quite consistent in both physiological and pathological status (Table 3).

### 3.5. Mechanism Investigation of the Varied Pharmacokinetic Characteristics

Liver takes an important role in the absorption, metabolism and elimination of most drugs, thus the liver status should greatly influence their pharmacokinetics. As expected, significant changes were observed in the plasma pharmacokinetic parameters of six compounds in YCHT in liver injured models as compared to in control. However, the mechanisms for existing phenomenon remain unknown.  Table 4 summarized the different changes of cytochrome P450s (CYP) in various liver diseases. From the table, we find that most liver diseases, including CCl_4_-induced liver injury, occurred accompanied with decreased activity or expression of some CYP1, CYP2, and CYP3, which could slow down the metabolism and increase bioavailabilities of absorbed compounds. And previous studies also demonstrated that the CCl_4_ intoxication resulted in the significant enhancement of many drugs' plasma exposures [16, 40]. Above factors may be favorable to explain the increased concentrations of the anti-injury agent in the plasma, and the desired effect can be obtained at lower doses.

In addition, hepatic drug transporters are also very important in pharmacokinetic features of therapeutic drugs. Disease states can often alter function of hepatic drug transporters. This could result in a change in systemic and/or tissue exposure of their substrates [41]. Okumura et al. reported that the hepatic transporters' expression changed in rats with liver dysfunction induced by CCl_4_ treatment, and the expression difference of transporters may affect the drug disposition and excretion [42]. Na^+^-taurocholate-cotransporting polypeptide, organic anion-transporting peptide-C, and organic cation transporter 1 showed remarkable decreases in the patients with chronic hepatitis C viral infection [43]. This may have some relationship with the change in absorption and metabolism of drugs.

The last possible factor is that P-glycoprotein (P-gp) activity can be systemically suppressed by CCl_4_-induced liver injury in mice, even though the level of P-gp stayed unchanged or rather increased [44]. As the function of P-gp is to pump foreign substances out of the cells, inhibition of P-gp activity could improve compounds' oral bioavailabilities.

However, as to emodin and scopoletin in YCHT, there are no changes in the plasma pharmacokinetic parameters between control and liver injured rats. The reason could be that the CYP enzymes were only partly changed during this kind of liver injury (Table 4). Whereas CYP enzymes, metabolizing emodin and scopoletin, may not be all altered after liver injury, which would lead to unchanged pharmacokinetics in liver injured rats as compared to in normal. In addition, emodin and scopoletin are not P-gp substrates [45, 46], so it would not be reduced because of the change of P-gp. Moreover emodin can also reverse CCl_4_-induced hepatic CYP enzymatic of hepatic drug-metabolizing enzymes [47], which could partly explain unchanged pharmacokinetic behavior. The causes and effects of the pharmacokinetic changes of components in YCHT still need further investigation.

## 4. Conclusion

In this study, a sensitive, selective and rapid UHPLC-MS based method was developed and validated for the quantitation of eight bioactive components of YCHT in normal and liver injured rats' plasma. The results demonstrated that hepatic injury could significantly influence the pharmacokinetics of scoparone, geniposide, aloe-emodin, rhein, chrysophanol, and physcion after oral administration of YCHT, except for scopoletin and emodin. For previous six ingredients, the metabolism and elimination processes slowed down and their bioavailabilities significantly increased in the model rats as compared with those in control. Above results indicated that liver injury would lead to a significant accumulation of bioactive compounds in YCHT during its treatment. We believe that our study would provide important references to realize better clinical applications of YCHT in liver injury. Moreover, our established method might be useful for other traditional Chinese formulas' research.

## Figures and Tables

**Table 1:** Precursor-product ion pairs and their voltage parameters of eight constituents and ISs.

Name	Mode	Precursor Ion (m/z)	MS1 Res	Product Ion (m/z)	MS2 Res	Dwell (ms)	Fragmentor (V)	CE (V)	Retention times (min)
Scoparone	+	207.1	Unit	107	Unit	100	155	41	2.85
Scopoletin	+	193.1	Unit	133	Unit	100	125	25	2.51
Glibenclamide (IS)	+	494.2	Unit	368.9	Unit	100	110	20	3.73
Geniposide	-	433	Unit	225	Unit	100	105	5	1.07
Aloe-emodin	-	269	Unit	240	Unit	100	135	25	1.90
Rhein	-	283	Unit	239	Unit	100	80	10	2.10
Emodin	-	269	Unit	225	Unit	100	150	30	2.75
Chrysophanol	-	253	Unit	225	Unit	100	160	30	3.36
Physcion	-	283	Unit	240	Unit	100	144	29	4.35
Wogonin (IS)	-	283	Unit	268	Unit	100	110	15	2.10

**Table 2:** Calibration curves, correlation coefficients, linear ranges, LLOD, and LOQ of eight constituents in rat plasma.

Analytes	Calibration curves	Correlation coefficient (r)	Linearity range (ng mL^−1^)	LLOD (ng mL^−1^)	LLOQ (ng mL^−1^)
Scoparone	*y*=6.7320*∗*10^−3^*x*+2.7130*∗*10^−3^	0.9984	0.510-2040.0	0.1020	0.5100
Scopoletin	*y*=1.1041*∗*10^−2^*x*+1.8124*∗*10^−2^	0.9990	0.502-50.2	0.1004	0.5020
Geniposide	*y*=1.7678*∗*10^−5^*x*+1.1606*∗*10^−5^	0.9985	0.516-2064.0	0.1032	0.5160
Aloe-emodin	*y*=3.5727*∗*10^−4^*x*-2.0007*∗*10^−4^	0.9987	0.532-106.4	0.1064	0.5320
Rhein	*y*=3.0210*∗*10^−3^*x*+1.9360*∗*10^−3^	0.9981	0.540-2160.0	0.1080	0.5400
Emodin	*y*=6.8730*∗*10^−3^*x*+1.4790*∗*10^−3^	0.9995	0.525-52.5	0.1050	0.5250
Chrysophanol	*y*=7.7718*∗*10^−5^*x*-3.0355*∗*10^−5^	0.9990	0.500-100.0	0.1000	0.5000
Physcion	*y*=9.7615*∗*10^−5^*x*-5.1742*∗*10^−5^	0.9987	0.512-51.2	0.1024	0.5120

**Table 3:** Pharmacokinetic parameters for eight compounds in plasma after oral administration of YCHT.

Pharmacokinetic Parameter		*t*_1/2_ (h)	C_max_ (*μ*g/L)	T_max_ (h)	AUC_0–t_ (ug/L*∗*h)	AUC_0–*∞*_ (ug/L*∗*h)	MRT_0–t_ (h)	MRT_0–*∞*_ (h)	CLz/F (L/h/kg)	Fr (%)
Scoparone	Control	2.14 ± 0.71	939.32 ± 112.19	0.54 ± 0.10	2282.77 ± 390.03	2347.92 ± 428.69	3.19 ± 0.16	3.51 ± 0.14	4.42 ± 0.82	227.64
	Model	1.58 ± 0.13	1767.17 ± 106.78*∗∗*	0.33 ± 0.13*∗*	5196.54 ± 513.37*∗∗*	5197.06 ± 513.33*∗∗*	3.94 ± 0.26*∗∗*	3.95 ± 0.26*∗∗*	1.96 ± 0.23*∗∗*	
Scopoletin	Control	3.62 ± 3.85	3.53 ± 0.67	0.19 ± 0.04	5.86 ± 0.50	7.41 ± 2.53	4.40 ± 0.44	6.90 ± 4.20	70.72 ± 16.41	123.38
	Model	2.20 ± 0.82	3.34 ± 1.19	0.13 ± 0.05*∗*	7.23 ± 1.84	7.83 ± 2.00	4.81 ± 0.43	5.56 ± 0.63	66.11 ± 16.51	
Geniposide	Control	3.55 ± 0.38	751.37 ± 47.72	0.33 ± 0.13	1940.63 ± 210.72	1958.65 ± 206.96	4.57 ± 0.41	4.80 ± 0.47	8.84 ± 0.92	257.67
	Model	6.59 ± 2.46*∗*	1863.84 ± 108.53*∗∗*	0.58 ± 0.20*∗*	5000.36 ± 755.13*∗∗*	5223.83 ± 746.92*∗∗*	4.68 ± 0.57	5.99 ± 0.61*∗∗*	3.34 ± 0.49*∗∗*	
Aloe-emodin	Control	2.26 ± 1.15	15.18 ± 1.68	0.54 ± 0.19	54.61 ± 6.50	60.86 ± 6.68	3.34 ± 0.12	4.33 ± 0.84	44.24 ± 5.84	281.30
	Model	1.98 ± 0.71	36.68 ± 3.09*∗∗*	0.58 ± 0.30	153.62 ± 18.13*∗∗*	158.71 ± 19.26*∗∗*	3.68 ± 0.34*∗*	4.07 ± 0.40	16.98 ± 2.15*∗∗*	
Rhein	Control	4.64 ± 2.14	663.62 ± 50.31	0.33 ± 0.13	2031.40 ± 385.58	2074.39 ± 387.77	4.79 ± 0.36	5.38 ± 0.49	4.92 ± 1.11	268.45
	Model	3.62 ± 1.97	1759.02 ± 140.49*∗∗*	0.54 ± 0.19*∗*	5453.29 ± 1202.14*∗∗*	5518.78 ± 1256.46*∗∗*	4.38 ± 0.41	4.67 ± 0.65	1.87 ± 0.43*∗∗*	
Emodin	Control	3.33 ± 1.67	3.51 ± 0.59	0.20 ± 0.17	10.41 ± 0.83	12.06 ± 1.53	4.94 ± 0.26	6.65 ± 1.99	69.69 ± 8.45	112.01
	Model	3.64 ± 0.79	3.89 ± 0.89	0.17 ± 0.05	11.66 ± 2.70	13.49 ± 2.43	4.83 ± 0.25	6.64 ± 0.78	63.22 ± 11.47	
Chrysophanol	Control	2.00 ± 0.23	34.48 ± 4.99	0.63 ± 0.26	99.34 ± 10.12	103.57 ± 10.09	2.93 ± 0.16	3.34 ± 0.21	21.80 ± 2.08	142.23
	Model	3.12 ± 1.74	37.61 ± 6.51	0.88 ± 0.35	141.29 ± 27.92*∗∗*	148.47 ± 25.04*∗∗*	3.43 ± 0.40*∗*	4.20 ± 0.77*∗*	15.42 ± 2.38*∗∗*	
Physcione	Control	2.65 ± 0.99	8.45 ± 1.61	1.25 ± 0.27	21.05 ± 4.04	22.44 ± 5.54	2.88 ± 0.25	3.54 ± 0.49	26.99 ± 5.66	213.68
	Model	3.06 ± 2.37	9.99 ± 2.86	2.58 ± 1.11*∗*	44.98 ± 13.92*∗∗*	48.47 ± 12.74*∗∗*	4.07 ± 0.40*∗∗*	5.26 ± 1.30*∗*	12.63 ± 3.12*∗∗*	

*∗* p<0.05 compared with control rats.

*∗∗* p<0.01 compared with control rats.

**Table 4:** The changes of CYP450s in various liver diseases.

Disease type	Organism	Change of CYP450s compared to control	Reference
Hepatocellular carcinoma	Human	The CYP2C9, CYP2D6, and CYP2E1 are increased. The CYP1A2, CYP2C8, and CYP2C19 activity decreased. And CYP2A6, CYP2B6, and CYP3A4/5 activity were unchanged.	[10]
Non-alcoholic fatty liver disease	Human	Hepatic CYP2E1 expression increased, and its activity was also up-regulated in the context of obesity and NAFLD	[11]
Viral hepatitis	Human	Levels of the CYPs were generally lower in CYP1A2, CYP2C19, and CYP2E1.	[12]
Liver cirrhosis	Human	The CYP1A and CYP3A levels and related enzyme activities are usually reduced. However, CYP2C, CYP2A, and CYP2B are mostly unaltered	[13, 14]
CCl_4_-induced liver injury	Mice	The expression of CYP2E1 is significant decreased.	[15]
CCl_4_-induced liver injury	Rat	The expression of CYP3A (CYP3A2) is decreased.	[16, 17]
CCl_4_-induced liver fibrosis	Rat	The mRNA level of CYP2E1 showed a significantly decreased.	[18]
CCl_4_-induced severe liver cirrhosis	Rat	The inducibility of CYP1A enzymes is well maintained in compensated cirrhosis, but it is markedly reduced when liver dysfunction becomes severe	[19]
Thioacetamide-induced liver cirrhosis	Rat	The hepatic protein expressions of CYP1A2, CYP2C6, CYP2E1, and CYP3A2 are dramatically reduced.	[20]
N-dimethyl nitrosamine-induced liver cirrhosis	Rat	The expression of CYP1A, 2B1/2, 2C11, 2D and 3A was significantly decreased,	[21, 22]

## Data Availability

The data used to support the findings of this study are available from the corresponding author upon request.

## Supplementary Materials

Supplementary Materials Figure S1. The chemical structures of eight major bioactive components from Yin Chen Hao Tang. Figure S2. The chemical structures of ISs. Table S1 Intra-day and inter-day precision and accuracy for eight compounds in rat plasma (n = 6). Table S2 Summary of matrix effect and recovery of the eight compounds for the UHPLC–MS/MS method in rat plasma (mean± SD, n = 6). Table S3 Stabilities of the analytes in plasma under various storage conditions (mean± SD, n = 5).
