# Corrigendum to “Development and Application of an UHPLC-MS/MS Method for Comparative Pharmacokinetic Study of Eight Major Bioactive Components from Yin Chen Hao Tang in Normal and Acute Liver Injured Rats”

**DOI:** 10.1155/2018/3024841

**Published:** 2018-12-16

**Authors:** Yun Wang, Xinrui Xing, Yan Cao, Liang Zhao, Sen Sun, Yang Chen, Yifeng Chai, Si Chen, Zhenyu Zhu

**Affiliations:** ^1^Hebei Institute for Drug Control, No. 16 Fuqiang Street, Shijiazhuang 050011, China; ^2^School of Pharmacy, Second Military Medical University, No. 325 Guohe Road, Shanghai 200433, China; ^3^Department of Pharmacy, Eastern Hepatobiliary Surgery Hospital, Shanghai 200438, China; ^4^Postdoctoral Research Workstation, 210th Hospital of the Chinese People's Liberation Army, Dalian 116021, China

In the article titled “Development and Application of an UHPLC-MS/MS Method for Comparative Pharmacokinetic Study of Eight Major Bioactive Components from Yin Chen Hao Tang in Normal and Acute Liver Injured Rats” [1], the Authors' Contributions section was missing and should be added as follows:

## Authors' Contributions

Yun Wang and Xinrui Xing contributed equally to this work.
