# Supplementary material for: Development and Application of an UHPLC-MS/MS Method for Comparative Pharmacokinetic Study of Eight Major Bioactive Components from Yin Chen Hao Tang in Normal and Acute Liver Injured Rats
Source: Evid Based Complement Alternat Med. 2018 Nov 1;2018:3239785. doi: 10.1155/2018/3239785 (PMC6241247; doi:10.1155/2018/3239785)
Supplement: Supplementary Materials — Figure S1. The chemical structures of eight major bioactive components from Yin Chen Hao Tang. Figure S2. The chemical structures of ISs. Table S1 Intra-day and inter-day precision and accuracy for eight compounds in rat plasma (n = 6). Table S2 Summary of matrix effect and recovery of the eight compounds for the UHPLC–MS/MS method in rat plasma (mean± SD, n = 6). Table S3 Stabilities of the analytes in plasma under various storage conditions (mean± SD, n = 5). [file 3239785.f1.doc]

**Supplemental Information for**

**Development and application of an UHPLC-MS/MS method for comparative pharmacokinetic study of eight major bioactive components from Yin Chen Hao Tang in normal and acute liver injured rats**

Yun Wang ^1^, Xinrui Xing ^2^, Yan Cao ^2^, Liang Zhao ^3^, Sen Sun ^3^, Yang Chen ^2^, Yifeng Chai ^2^, Si Chen ^4^,Zhenyu Zhu ^2^

^1^ Hebei Institute for Drug Control, No.16 Fuqiang Street, Shijiazhuang 050011, PR China

^2^ School of Pharmacy, Second Military Medical University, No. 325 Guohe Road, Shanghai 200433, PR China

^3^ Department of Pharmacy, Eastern Hepatobiliary Surgery Hospital, Shanghai 200438, PR China

^4^ Postdoctoral Research Workstation, 210th Hospital of the Chinese People’s Liberation Army, Dalian 116021, PR China

Correspondence should be addressed to Si Chen; caroline-sisi-chen@hotmail.com and Zhenyu Zhu; zzyzyfzhu@163.com

**Figures**


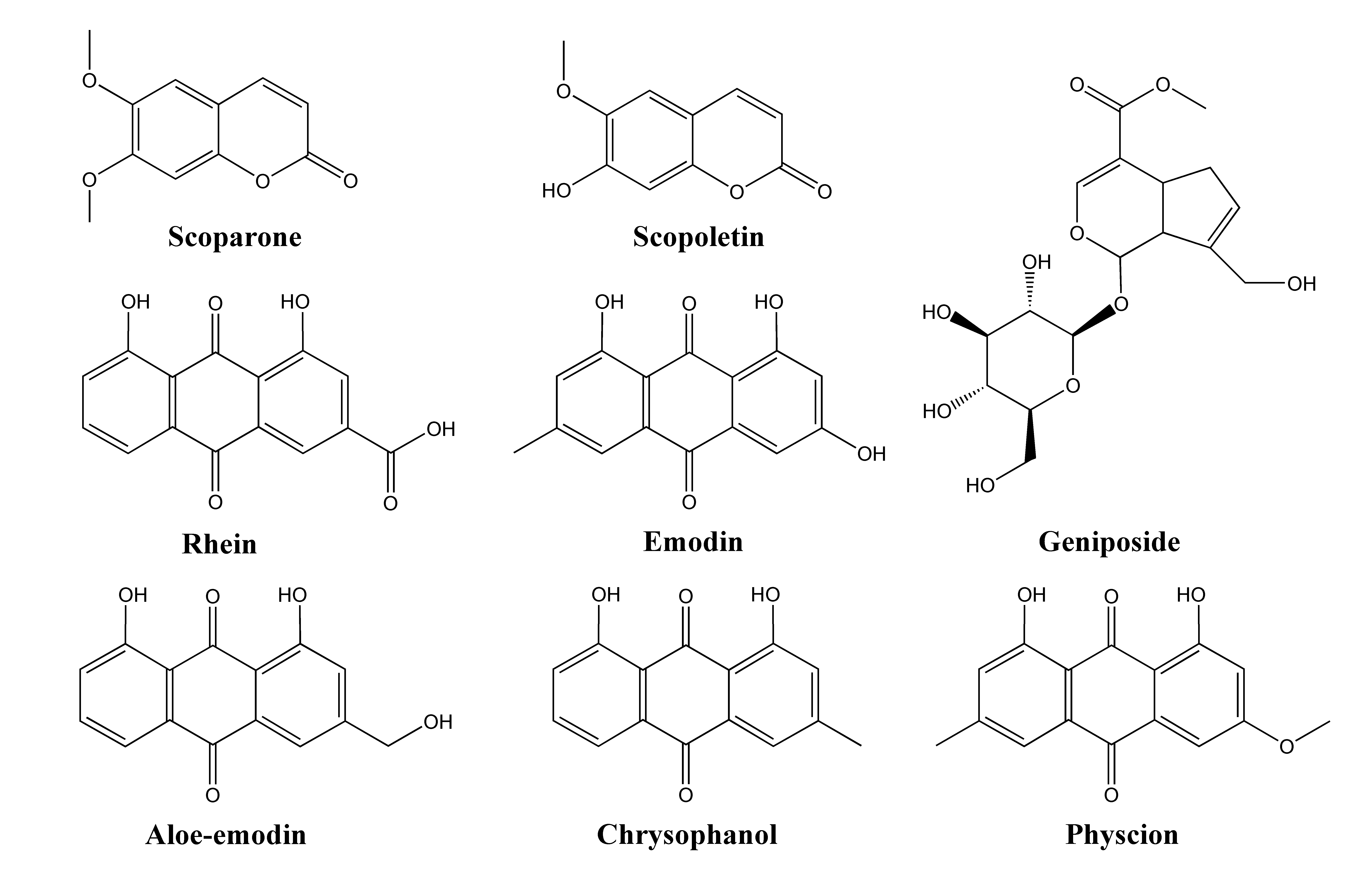


**Figure S1.** The chemical structures of eight major bioactive components from Yin Chen Hao Tang.


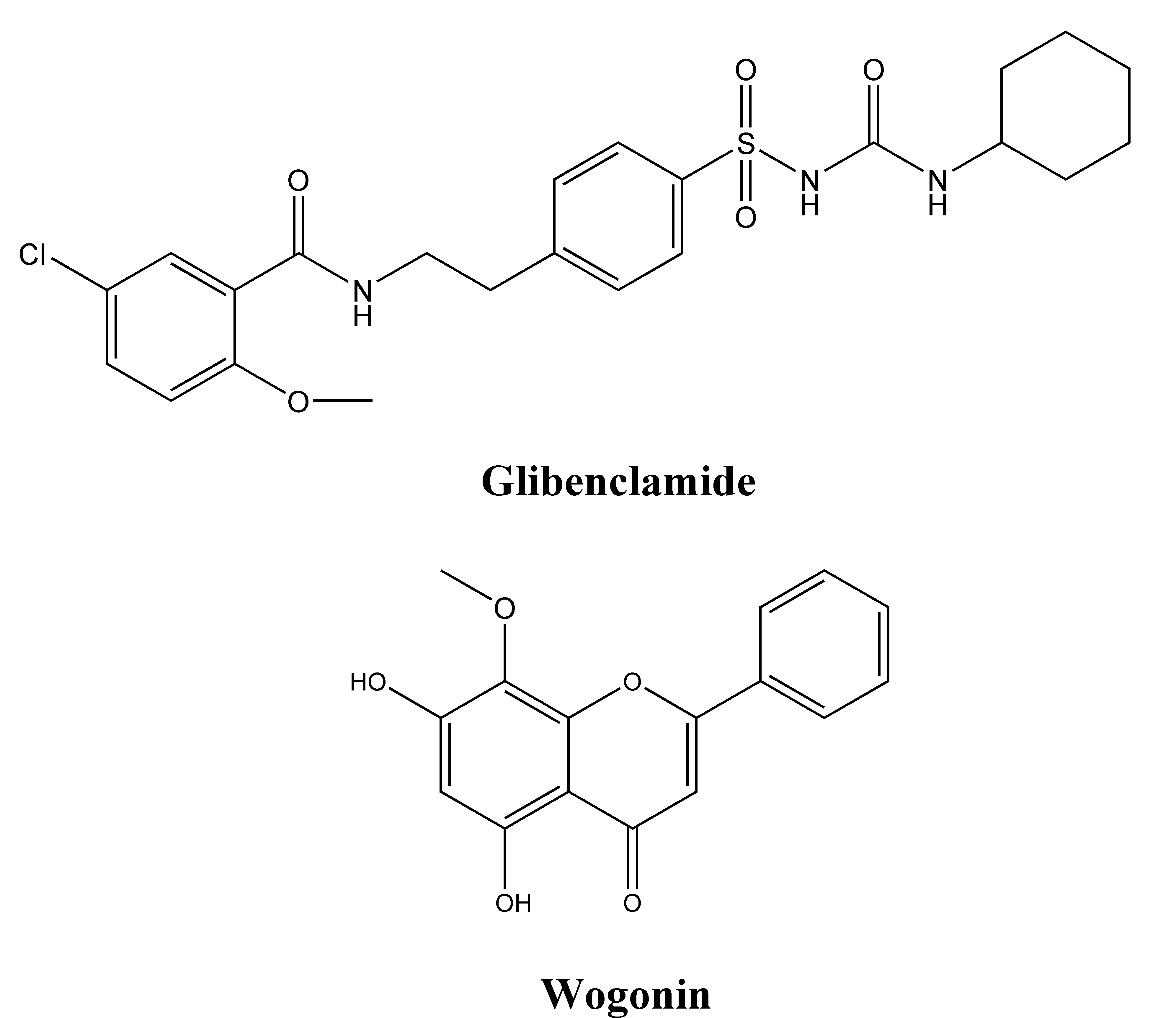


**Figure S2**. The chemical structures of ISs.

**Tables**

**Table S1** Intra-day and inter-day precision and accuracy for eight compounds in rat plasma (n = 6).

| Analytes | Concentration added (ng mL^-1^) | Intra-day precision (RSD, %) | Inter-day precision (RSD, %) | Accuracy (RE, %) |
| --- | --- | --- | --- | --- |
| Scoparone | 1.02 | 3.86 | 6.92 | -0.52 |
|  | 510.00 | 5.56 | 4.32 | 1.06 |
|  | 1530.00 | 2.48 | 4.16 | -0.86 |
| Scopoletin | 1.00 | 9.55 | 8.88 | -4.75 |
|  | 10.04 | 6.87 | 5.55 | 2.46 |
|  | 40.16 | 6.20 | 4.38 | -8.96 |
| Geniposide | 1.03 | 5.34 | 4.53 | -9.28 |
|  | 516.00 | 2.89 | 3.59 | -5.48 |
|  | 1548.00 | 1.51 | 1.57 | -2.99 |
| Aloe-emodin | 1.06 | 2.75 | 3.21 | -5.58 |
|  | 21.28 | 2.76 | 3.09 | -6.28 |
|  | 85.12 | 3.30 | 3.20 | -5.93 |
| Rhein | 1.08 | 6.07 | 5.10 | -1.69 |
|  | 540.00 | 4.09 | 4.03 | -2.36 |
|  | 1620.00 | 5.12 | 3.74 | -1.73 |
| Emodin | 1.05 | 4.54 | 6.59 | -1.15 |
|  | 10.50 | 5.39 | 5.09 | -2.71 |
|  | 42.00 | 3.64 | 3.80 | -1.25 |
| Chrysophanol | 1.00 | 5.47 | 6.87 | -1.86 |
|  | 20.00 | 4.57 | 4.08 | -1.38 |
|  | 80.00 | 6.01 | 4.31 | 1.44 |
| Physcion | 1.02 | 3.83 | 5.11 | -10.72 |
|  | 10.24 | 6.20 | 5.96 | -3.54 |
|  | 40.96 | 4.27 | 3.90 | -2.27 |

**Table S2** Summary of matrix effect and recovery of the eight compounds for the UHPLC–MS/MS method in rat plasma (mean± SD, n = 6).

| Analytes | Concentration added (ng/mL) | Matrix effect (%, mean±S.D.) | RSD(%) | Recoveries  (%, mean±S.D.) | RSD(%) |
| --- | --- | --- | --- | --- | --- |
| Scoparone | 1.02 | 91.11±4.00 | 4.39 | 88.84±1.88 | 2.12 |
|  | 510.00 | 94.85±4.36 | 4.60 | 90.92±1.72 | 1.89 |
|  | 1530.00 | 103.95±2.15 | 2.07 | 98.78±3.61 | 3.65 |
| Scopoletin | 1.00 | 99.63±7.70 | 7.73 | 104.74±4.70 | 4.49 |
|  | 10.04 | 98.97±3.19 | 3.22 | 95.96±3.05 | 3.18 |
|  | 40.16 | 104.96±4.33 | 4.13 | 106.49±5.30 | 4.98 |
| Geniposide | 1.03 | 97.53±5.12 | 5.25 | 88.39±5.62 | 6.36 |
|  | 516.00 | 92.89±4.16 | 4.48 | 98.36±1.47 | 1.49 |
|  | 1548.00 | 88.28±1.74 | 1.97 | 102.10±3.22 | 3.15 |
| Aloe-emodin | 1.06 | 92.51±4.31 | 4.66 | 95.02±3.43 | 3.61 |
|  | 21.28 | 86.74±4.16 | 4.80 | 105.76±3.51 | 3.32 |
|  | 85.12 | 91.35±3.76 | 4.12 | 96.77±3.12 | 3.22 |
| Rhein | 1.08 | 105.15±3.48 | 3.31 | 101.71±3.69 | 3.63 |
|  | 540.00 | 96.21±2.58 | 2.68 | 93.71±3.68 | 3.93 |
|  | 1620.00 | 89.44±4.47 | 5.00 | 96.17±3.54 | 3.68 |
| Emodin | 1.05 | 110.66±3.19 | 2.88 | 105.15±3.84 | 3.65 |
|  | 10.50 | 111.30±2.26 | 2.03 | 107.35±4.42 | 4.12 |
|  | 42.00 | 107.90±3.66 | 3.39 | 110.72±1.56 | 1.41 |
| Chrysophanol | 1.00 | 108.71±4.00 | 3.68 | 102.63±7.06 | 6.88 |
|  | 20.00 | 107.35±6.26 | 5.83 | 109.18±3.03 | 2.78 |
|  | 80.00 | 106.84±4.87 | 4.56 | 97.51±3.79 | 3.89 |
| Physcion | 1.02 | 102.72±5.00 | 4.87 | 99.02±4.43 | 4.47 |
|  | 10.24 | 99.41±3.99 | 4.01 | 105.65±3.54 | 3.35 |
|  | 40.96 | 110.67±2.97 | 2.68 | 96.23±4.66 | 4.84 |

**Table S3** Stabilities of the analytes in plasma under various storage conditions (mean± SD, n = 5).

| Analytes | Added concentration (ng/ml) |  | Room temperature for 2h (Bench top) | |  | | Frozen (-80℃) for 15 days | | |  | | Three freeze-thaw cycles at -80℃ | | |  | | Post-pretreatment at 4℃ for 24h | | |  |
| --- | --- | --- | --- | --- | --- | --- | --- | --- | --- | --- | --- | --- | --- | --- | --- | --- | --- | --- | --- | --- |
|  |  |  | Measured concentration (ng/ml) | RSD（%） | |  | | Measured concentration (ng/ml) | RSD（%） | |  | | Measured concentration (ng/ml) | RSD（%） | |  | | Measured concentration (ng/ml) | RSD（%） | |
| Scoparone | 1.02 |  | 1.05±0.06 | 5.71 | |  | | 1.07±0.07 | 6.54 | |  | | 1.03±0.09 | 8.74 | |  | | 0.96±0.06 | 6.25 | |
|  | 510.00 |  | 542.75±7.35 | 1.35 | |  | | 540.29±27.49 | 5.09 | |  | | 492.02±15.56 | 3.16 | |  | | 536.94±29.08 | 5.42 | |
|  | 1530.00 |  | 1506.14±76.72 | 5.09 | |  | | 1492.30±31.19 | 2.09 | |  | | 1457.75±52.46 | 3.60 | |  | | 1590.10±88.05 | 5.53 | |
| Scopoletin | 1.00 |  | 0.94±0.06 | 6.38 | |  | | 1.08±0.07 | 6.48 | |  | | 1.07±0.07 | 6.54 | |  | | 1.04±0.06 | 5.77 | |
|  | 10.04 |  | 10.21±0.42 | 4.11 | |  | | 10.12±0.57 | 5.63 | |  | | 9.62±0.75 | 7.80 | |  | | 10.36±0.51 | 4.92 | |
|  | 40.16 |  | 37.31±1.22 | 3.27 | |  | | 37.79±1.72 | 4.55 | |  | | 36.90±1.25 | 3.39 | |  | | 37.28±1.74 | 4.67 | |
| Geniposide | 1.03 |  | 0.95±0.05 | 5.26 | |  | | 0.96±0.03 | 3.12 | |  | | 0.95±0.05 | 5.26 | |  | | 0.99±0.04 | 4.04 | |
|  | 516.00 |  | 479.74±8.69 | 1.81 | |  | | 486.35±6.08 | 1.25 | |  | | 482.76±12.14 | 2.51 | |  | | 478.00±22.74 | 4.76 | |
|  | 1548.00 |  | 1420.27±41.79 | 2.94 | |  | | 1434.24±60.84 | 4.24 | |  | | 1431.52±45.66 | 3.19 | |  | | 1443.33±37.83 | 2.62 | |
| Aloe-emodin | 1.06 |  | 1.01±0.05 | 4.95 | |  | | 0.98±0.02 | 2.04 | |  | | 0.98±0.02 | 2.04 | |  | | 1.02±0.05 | 4.90 | |
|  | 21.28 |  | 20.82±0.28 | 1.34 | |  | | 20.94±0.27 | 1.29 | |  | | 20.01±0.29 | 1.45 | |  | | 20.29±0.89 | 4.39 | |
|  | 85.12 |  | 81.50±1.56 | 1.91 | |  | | 80.57±2.29 | 2.84 | |  | | 80.66±2.40 | 2.98 | |  | | 81.80±2.56 | 3.13 | |
| Rhein | 1.08 |  | 1.06±0.06 | 5.66 | |  | | 1.06±0.03 | 2.83 | |  | | 1.02±0.05 | 4.90 | |  | | 1.07±0.03 | 2.80 | |
|  | 540.00 |  | 473.38±6.00 | 1.27 | |  | | 490.13±8.26 | 1.69 | |  | | 488.08±16.62 | 3.41 | |  | | 492.24±4.64 | 0.94 | |
|  | 1620.00 |  | 1462.55±40.60 | 2.78 | |  | | 1461.33±35.01 | 2.40 | |  | | 1408.54±39.89 | 2.83 | |  | | 1463.44±50.48 | 3.45 | |
| Emodin | 1.05 |  | 0.95±0.05 | 5.26 | |  | | 1.10±0.04 | 3.64 | |  | | 0.97±0.09 | 9.28 | |  | | 1.02±0.08 | 7.84 | |
|  | 10.50 |  | 10.80±0.35 | 3.24 | |  | | 9.45±0.34 | 3.60 | |  | | 9.60±0.19 | 1.98 | |  | | 9.88±0.63 | 6.38 | |
|  | 42.00 |  | 41.95±2.55 | 6.80 | |  | | 39.01±2.32 | 5.95 | |  | | 40.62±2.31 | 5.69 | |  | | 38.82±1.08 | 2.78 | |
| Chrysophanol | 1.00 |  | 1.00±0.05 | 5.00 | |  | | 0.98±0.06 | 6.12 | |  | | 1.00±0.11 | 11.00 | |  | | 1.06±0.07 | 6.60 | |
|  | 20.00 |  | 21.21±1.83 | 8.63 | |  | | 21.12±0.58 | 2.75 | |  | | 20.46±1.15 | 5.62 | |  | | 21.87±1.19 | 5.44 | |
|  | 80.00 |  | 78.10±3.36 | 4.30 | |  | | 75.85±3.98 | 5.25 | |  | | 83.81±3.51 | 4.19 | |  | | 81.55±3.42 | 4.19 | |
| Physcion | 1.02 |  | 0.94±0.04 | 4.26 | |  | | 0.91±0.02 | 2.20 | |  | | 0.92±0.05 | 5.43 | |  | | 0.92±0.04 | 4.35 | |
|  | 10.24 |  | 9.37±0.42 | 4.48 | |  | | 9.98±0.30 | 3.01 | |  | | 9.19±0.35 | 3.81 | |  | | 9.65±0.69 | 7.15 | |
|  | 40.96 |  | 37.96±1.51 | 3.98 | |  | | 38.60±1.16 | 3.01 | |  | | 37.77±1.41 | 3.73 | |  | | 38.23±1.49 | 3.90 | |
